# Assessing the impact of Covid-19 on nurturing care in Nairobi slums: Findings from 5 rounds of cross-sectional telephone surveys

**DOI:** 10.1371/journal.pgph.0003286

**Published:** 2025-05-28

**Authors:** Robert C. Hughes, Silas Onyango, Nelson Langat, Ruth Muendo, Rachel Juel, Elizabeth Kimani-Murage, Zelee Hill, Betty Kirkwood, Sunil S. Bhopal, Patricia Kitsao-Wekulo

**Affiliations:** 1 Department of Population Health, London School of Hygiene & Tropical Medicine, London, United Kingdom; 2 Maternal and Child Wellbeing Unit, African Population and Health Research Center, Nairobi, Kenya; 3 Department of Epidemiology and Public Health, University College London, London, United Kingdom; 4 Bradford Institute for Health Research, Bradford, United Kingdom; NYU Grossman School of Medicine: New York University School of Medicine, UNITED STATES OF AMERICA

## Abstract

The Covid-19 pandemic and mitigation measures had widespread societal impacts. Young children are particularly vulnerable yet the ways the pandemic affected children in informal settlements (slums) are not well described. This study aimed to investigate the impacts of the pandemic on early childhood in three Nairobi informal settlements. Five rounds of cross-sectional computer assisted telephone surveys (with 578–774 respondents in each) in three settlements (Kibera; Mukuru-Viwandani; Kawangware) over 13 months, correlating with different phases Covid-19 restrictions. Impact was assessed through comparing changes in summary statistics on responses to survey questions on each domain of Nurturing Care over time. Survey results found significant disruptions in healthcare services, particularly in early rounds with missed vaccinations (reported by 1 in 5 parents of infants) and therapeutic healthcare seeking (missed by up to 21% of families). Persistent food and nutrition insecurity, with a large majority of families struggling to feed their children (72% in Round 1) due to financial constraints. Economic shocks were near-universal; 99.7% of respondents reporting earning less since the start of the pandemic. Use of paid childcare initially plummeted but showed a resurgence over time (up to 21% by Round 5) as pandemic control measures evolved. Young children were commonly left alone in all rounds, but especially earlier ones; underscoring the enduring challenges in providing nurturing care in these settings. Very few (<2%) of study participants reported direct experience of illness from Covid-19 in their family in any round. In conclusion, despite adaptations over time and the decrease in reported disruptions, prolonged economic shock was associated with multiple adverse effects on Nurturing Care. The study’s longitudinal scope provides insights into the dynamic nature of ensuring young children in slums thrive during crises, highlighting the need for interventions and policies that address the compounded vulnerabilities of young children in these communities.

## 1 Introduction

Early childhood development (ECD), by which we refer to the first five years of a child’s life, is central to setting a child’s developmental trajectory. This period is a critical one for accumulation or loss of ‘human capital’; it is a period when adversities can have lasting impacts on rapidly developing brains, impacting on learning, later-life earning and overall wellbeing [1]. In recognition of this, the early years have received increasing attention from policy makers [2], funders [3,4] and academics [5] in recent years. This culminated in the launch of the Nurturing Care Framework by the WHO/UNICEF and World Bank in 2018. This Framework attempts to capture the key ‘components’ or domains of what is needed for a child to thrive in the early years, and includes the following five components: Health; Nutrition; Responsive Caring; Early Learning; and Security and Safety [2].

However, there is limited published literature on ECD in urban areas, particularly in slums or informal settlements, despite the rapid growth of these settings. In particular, little is known about either the provider of care, or what care is provided to young children [6]. In addition, the SARS-Cov-2 pandemic from 2020 led to radical disruption of early childhood in almost every country around the world due to both the direct and the indirect effects of the virus and efforts to control it, efforts which frequently included strict ‘stay at home’ orders or ‘lockdowns’, with broad and deep impacts across societies as workplaces, nurseries, schools and public spaces were closed or restricted [7]. While there has been considerable emphasis and research into the effects of the pandemic on health systems and services [8], there has been little work on early childhood specifically, especially in low- and middle-income countries where resilience to radical disruption may be most limited [9]. It is also instructive to consider the pre-pandemic context in Kenya, where 36% of the population have an income of less than US$2.15 a day [10]. Data from recent research in Nairobi suggested that for 46% of employed mothers, and 23% of unemployed mothers, regularly use paid childcare [11].

We aimed to track over time how the care of young children in three slums in Nairobi was affected by the pandemic and efforts to control it. We aimed to estimate impacts across all five components of Nurturing Care (Health, Nutrition, Responsive Caring, Early Learning and Security and Safety) alongside cross-cutting impacts(for example household economic impacts) during a year of the pandemic. This study is nested within the broader Nairobi Early Childhood Care in Slums (NECS) study, a detailed mixed-methods exploration of the care of children in Nairobi slums [12].

Our key pre-study hypotheses were: firstly, that the evolving Covid-19 pandemic would be likely to impact on Nurturing Care in slums, and that these impacts would be felt across multiple domains. Secondly, we hypothesised that these impacts may evolve over time, in relation to both Covid-19 case incidence and the stringency of epidemic control measures in force.

## 2 Methods

### 2.1 Setting

The setting for the study was three slums in Nairobi, Kenya: Kibera; Mukuru-Viwandani; and Kawangware. These slums are characterized by high levels of poverty, poor sanitation, inadequate shelter, poor infrastructure, high levels of insecurity, and low rates of formal employment [13,14]. These slums were selected for several reasons: firstly, these characteristics meant that they represented a particularly vulnerable group; secondly, because of access to a pre-existing database of residents who had consented to being contacted for surveys (described further in section 2.6); and thirdly, to overlap with the wider Nairobi Early Childcare in Slums Study (NECS) [12].

### 2.2 Study design

As part of a longitudinal design, five rounds of cross-sectional surveys were undertaken through computer-assisted telephone interviews (CATI). Participants were parents of children aged under five years selected from a pre-existing database of around 48,000 low-income household contacts living in three Nairobi slums (Kibera (c. 25,000 contacts), Kawangware (c. 13,000 contacts) and Mukuru-Viwandani (c. 10,000 contacts). These were people who had previously participated in surveys/field experiments conducted by the non-profit research firm BUSARA [15] and who had consented to being invited to participate in future studies. These potential participants were initially recruited door-to-door by field assistants.

**Fig 1:**
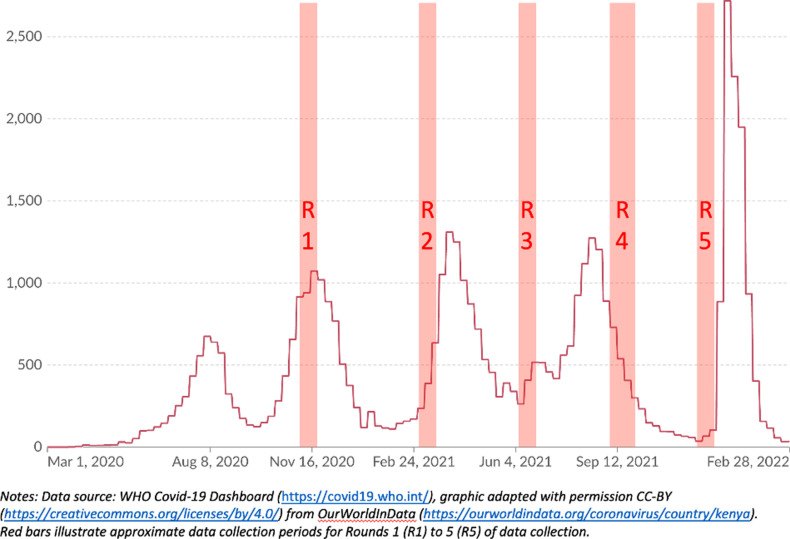
Graph illustrating timing of data collection in relation to 7-day rolling average numbers of confirmed Covid-19 cases in Kenya.

The first round of data collection was from 10 to 29 November 2020, which corresponded with the peak of Kenya’s second wave of recorded Covid-19 infections. Rounds 2–5 were all in 2021 (Round 2: data 9–29 March; Round 3: 6–25 June; Round 4: 6 September -1 October; Round 5: 29 November-13 December). Fig 1 and Table 1 summarises the prevailing Covid-19 epidemiology (and controls) in place at the time of each round of data collection.

**Table 1:** The context for each survey round.

Round	1	2	3	4	5
**Data collection dates:**	10^th^ -29^th^ November 2020	9^th^-29th March 2021	6^th^-25^th^ June 2021	6^th^ September-1^st^ October 2021	29^th^ November-13^th^ December 2021
**Season**	*Short rains*	*Start of long rains*	*Dry – start of cold season*	*Start of short rains*	*End of short rains*
**Summary of covid epidemiology at the time –** narrative; overall trend in epidemiology	*This period coincided with* ***the peak of Kenya’s second wave of recorded Covid-19 infections****, which peaked in mid-November. Towards the end of the data collection period cases had begun to fall.*	*A period of high growth in recorded Covid-19 case; during this period the* ***Kenya’s third wave of infections*** *was emerging. The wave peaked towards the end of this round of data collection.*	*A period of* ***relatively low Covid-19 cases*** *compared to previous rounds, albeit with some growth in recorded cases after the data collection period.*	***A period of falling Covid-19 cases*** *, after the peak, in mid-August, of the country’s fourth wave.*	***Very low levels of cases of Covid-19 recorded*** *, the lowest since the start of the pandemic. Towards the end of the data collection period some rise in cases which turned out to be the start of the fifth wave in Kenya.*
**Mean daily covid cases (across data collection period)**.^*^	1021	1074	518	388	97
**Range in daily cases**	413-1459	431-2008	161–796	54–704	28–202
**Covid control measures in place:**					
**Curfew**	Yes – 10pm-4am	Yes – 8pm-4am	Yes – 8pm-4am	Yes – 10pm–4am	No
**Ban on gatherings**	Yes – all types	Yes – all types	Yes – all types	Limited – no more than 15, except weddings and funerals (up to 100)	Unrestricted
**Public messaging**	*‘’No Mask, No service”* *“Stay at home”*	*“Work from home if you can”*	*“Work from home if you can”*	*“Mask mandatory in public places”* *“Get vaccinated”*	*“Return to normal”*
**Closure of hospitality**	Yes	Yes	Yes	Partial	No
**Schools**	*Mostly closed – staggered re-opening for some grades*	*Partially closed – exam grades in school for delayed exams*	*Open for all grades*	*Open for all grades*	Open for all grades
**Covid vaccination rollout**	*No vaccine available*	*Vaccine rollout being planned, but not yet rolling out.*	*Mass vaccination programme rolled out, around about one million vaccinated.*	*Mass vaccination campaign ongoing; 3.8 m people have received at least one dose.*	*5 million people have received at least one dose, 3.3m have received two doses; Proportion of adults fully vaccinated was 12.0%.*
**Covid stringency Index – mean (range)**	51.9 (51.85–51.85)	52.7 (50.93–64.35)	66.2 (65.74–74.07	56.0 (56.02–56.02)	41.4 (41.33–41.39)

*Data obtained from the Ministry of Health official website *https://www.health.go.ke/press-releases/*

± See COVID-19 Government Response Tracker | Blavatnik School of Government (ox.ac.uk)

### 2.3 Sampling and recruitment

Survey respondents were selected randomly from the BUSARA database. The recruitment procedure was as follows:

1. A randomly ordered list of potentially eligible respondents was identified based on their being resident in the three slum areas (52% of these were from Kibera, 27% Kawangware, and 21% from Mukuru-Viwandani);
2. An SMS message was sent briefly introducing the research and informing them that they would be called.
3. An eligibility check telephone call was completed, during which, if eligible and interested, the background and rationale for the study were explained, followed by a clear and explicit consent process. This was followed by either immediate completion of survey, or a further scheduled callback at convenient time

Up to five call attempts were made to reach each potential participant each round, at a variety of times during the day. When surveys were interrupted by a dropped call or other interruption, up to five attempts were made to complete the survey over the following days.

Steps 2–3 were repeated in each subsequent round of data collection. If a round was missed, respondents remained in the study, and the same procedures applied in each subsequent round.

### 2.4 Respondent eligibility criteria

Respondents were eligible to participate in the study if they: (i) were 18 years or older (self-reported, with no formal verification); (ii) had a child aged under 5 years old living in their households (or who lived there at some point in the year 2020; in order to avoid excluding children who had moved out of the city due to the pandemic); (iii) were resident in the study setting. In addition, individuals needed to have provided consent to BUSARA within the last 5 years to be contacted with invitations for future research studies.

### 2.5 Data collection

Data were collected by enumerators working for the non-profit research firm BUSARA [15] who had expertise in public health and epidemiology research. The SurveyCTO [16] platform was used for digital data collection which was used to guide call-centre operators, (who were experienced in survey work), through the survey questions in Kiswahili. Enumerators had been trained for a total of 12 hours before the start of data collection. They worked from home (due to Covid-19 control measures) with daily check-in video call meetings with supervisors. Data quality was managed through enumerator training and pre-testing, automated range and consistency checks, manual checks on a sample of the data, debriefings, and refresher training. Survey interviews were digitally audio recorded for quality checks (conducted by supervisors on 10% of total surveys in each round).

### 2.6 Survey content

The survey consisted of a set of mostly closed questions. The survey questions were derived from a previously developed conceptual framework and draft household survey tool for the Nairobi Early Childcare in Slums Study [12], supplemented with additional questions aiming to identify what we considered might be both direct and indirect impacts of the Covid-19 pandemic on young children and their families.

Questions aimed to elicit signals of these impacts, and covered the following domains:

- Respondent characteristics and survey eligibility
- Child characteristics, including age, and reported sensory or mobility difficulties
- Who was looking after their youngest child, for even a few minutes and for >1 hour in the previous three days.
- Additional questions about the usage frequency and costs of paid childcare amongst those reporting using it
- The Family Care Indicators (FCI) questions about daily activities [17]
- Disruptions to healthcare services or care seeking
- Food and nutrition security, including impacts on breastfeeding
- Perceptions of community safety and security and reports of violence against children
- Levels of concern about the pandemic
- Economic impacts of the pandemic, including loss of work or income
- Help received
- Household assets (only the first time respondents took part)

Where there was more than one child in the house, respondents were asked to focus on the youngest child in the household. S1 Appendix includes the full survey instruments for Round 1 and Rounds 2–5, noting the minor changes that were added following review of Round 1 data quality and free text ‘other’ answers.

### 2.7 Sample size

In Round 1 we planned to recruit an initial 600 participants, allowing for a 20% loss to follow up, with the aim of achieving 480 participants who were followed throughout the study. This was calculated in order to yield estimates of the type of childcare used with precisions of at most + /- 5% including a 25% adjustment for clustering. In practice, we oversampled (by 174, to a total of 774 responses) in Round 1, to allow for higher levels of loss to follow up. In Rounds 2–5, new respondents were recruited following the same procedures as in Round 1.

### 2.8 Data analysis

**Fig 2:**
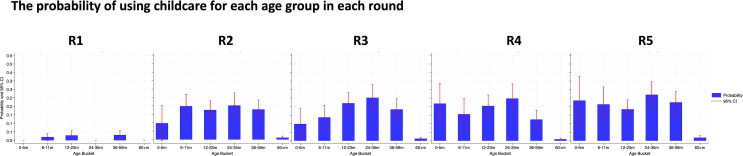
The probability of using paid childcare for each age group in each round.

**Fig 3:**
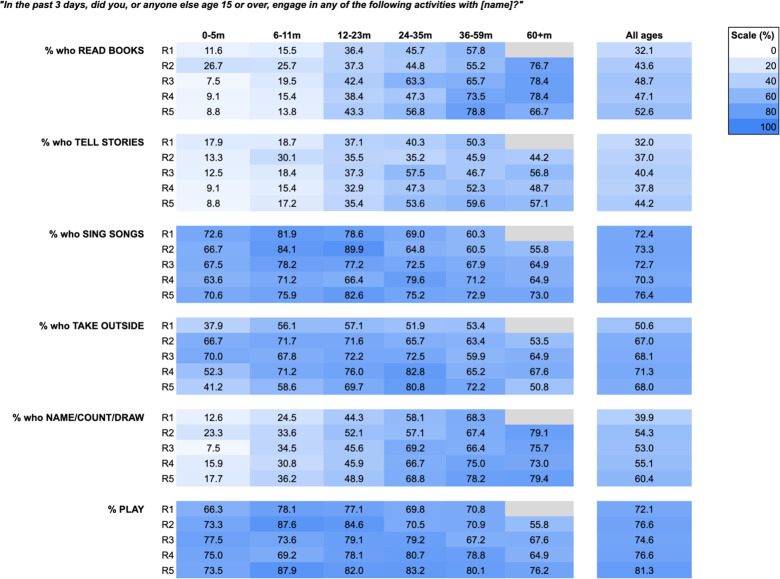
Heatmap illustrating % of respondents reporting ‘yes’ to family care indicators by age.

**Fig 4:**
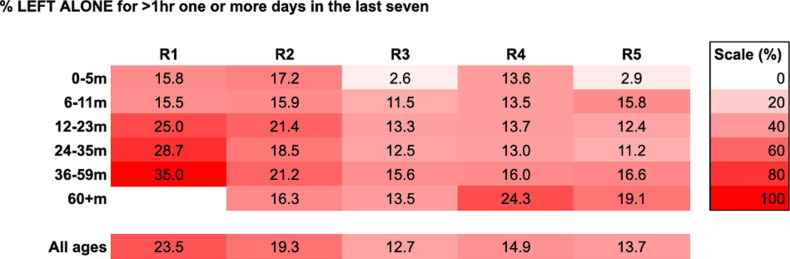
Heatmap of % of children reported to be left alone by age group and round.

Data were analysed using STATA 18 [18] and Microsoft Excel365 [19]. Descriptive summary statistics were calculated to describe the data for each round and changes between rounds. The frequency of reported impacts across all domains of Nurturing Care and cross-cutting impacts was reported with some responses (breastfeeding problems, the use of childcare, FCI indicators and children left alone) disaggregated by age of child as per Table 3 and Figs 2-4. A principal component analysis (PCA) was conducted based on a list of 21 reported household assets to generate socio-economic status (SES) quintiles for each unique respondent. Only surveys that were completed fully were shared by BUSARA; i.e., there were no missing data in the dataset we received.

### 2.9 Public involvement

During the design and inception phase for the wider NECS Study (in February 2020), public engagement meetings were held with community-based organisations in Mukuru-Viwandani. These meetings provided an opportunity to discuss the research methods and questions with the local community, including the planned household survey that informed these telephone surveys. Pre-study visits to the study site allowed for initial discussions about the research questions with parents, childcare providers, and other community members. During preparation of this manuscript emerging draft findings were shared in a community meeting in Nairobi in March 2022, where these findings were discussed with community members, with a focus on implications of the research for local planning and policy making.

### 2.10 Ethics

Informed verbal consent was obtained from all respondents and was recorded for audit purposes. The consent process included provision of information about the purpose of the study, confidentiality, how data would be used and shared, and the right to withdraw consent at any time. Participants who completed a survey round received KES150 (equivalent to US$1.5) for each round of survey completed to compensate them for their costs of participation). The LSHTM Research Ethics Committee (LSHTM Ref: 22692) and Amref Health Africa’s Ethics and Scientific Review Committees (ESRC) in Kenya (Ref: P777/2020) reviewed and approved the study protocol. The National Commission for Science, Technology and Innovation (NACOSTI) provided research clearance. The consent script and anticipated ‘frequently asked questions’ are included in S2 Appendix.

### 2.11 Role of the funders

The NECS Study was funded by The British Academy (Grant ECE190134) and Echidna Giving who supported a linked Clinical Research Fellowship for RCH. Echidna Giving also provided a ‘Covid Response’ grant for additional costs associated with the Covid-impacts tracker study that is reported on in this manuscript. The funders had no role in the study design, data collection, data analysis, data interpretation, or writing of the paper. The corresponding author had full access to all the data in the study and had final responsibility for the decision to submit for publication.

## 3 Results

### 3.1 Survey and respondent characteristics by round

A total of 1077 participants completed at least one round (774 respondents in R1, 664 in R2, 594 in R3, 528 in R4 and 642 in R5). The flow between rounds is illustrated in S1 Fig, a Sankey diagram illustrating flow and loss between survey rounds, which shows that 774 entered the study at R1; 122 at R2, 58 at R3, 62 at R4, 61 at R5, and that most loss to follow up occurred between R1 and R2. 279 respondents completed all 5 rounds (197 completed 4 rounds; 131 three rounds; 134 two rounds; 336 one round) (S1 Tables).

The median duration of interviews in R1 was 27 minutes, which increased to 34 minutes in R2 (when additional questions were added), and then reduced to 23, 20 and then 17 mins in subsequent rounds (as the number of new recruits fell and familiarity with the questions increased, in addition, questions about household assets were only asked in the first round that a respondent took part in). Most respondents were interviewed on the first call attempt (86–95% in R2-5; data on the number of call attempts was not retained by BUSARA in R1) (S1 Tables).

Table 2 summarises the respondent characteristics by round. Respondents were mostly mothers (52%), fathers (35%) or grandmothers (8%) (see S1 Tables). The mean age of survey respondents was similar across rounds (Range across rounds: 32.0 to 33.8 years old), as was the percent educated to 18 years old or more (Range: 28.3% to 33.4%). Similarly, the proportion of respondents in the lowest socio-economic quintile was consistent across rounds (Range: 20.5% to 17.4%).

**Table 2:** Survey respondent characteristics by round.

Round	1	2	3	4	5
n=	774	664	594	528	642
Of whom newly recruited	774	122	58	62	61
**Age of survey respondent** (yrs): Mean	32.0	32.9	33.2	33.0	33.8
Median	31	31	32	32	32
Range	19–67	19–67	20–56	19–67	18–88
**% educated to age 18yrs+**	-*^§^	33.4%	32.9%	32.3%	28.3%
n	*	222	196	171	182
**Mean age of participant’s youngest child** (months):	*21.0* ^±^	31.3	28.5	31.3	31.5
Inter-quartile range of ages of youngest child: (SD)	*30 (18.3)*	35 (22.0)	29 (21.6)	30 (21.6)	30 (19.6)
**SES Status** – % in lowest quintile	20.5	18.1	18.5	17.4	18.2
**Reported disabilities:** % parents report their youngest child having a problem with any of: seeing; hearing; moving; communicating	8.9%	9.3%	10.1%	9.6%	8.7%
n	69	62	60	51	56

§This question was asked differently after Round 1, as there appeared to be some confusion in how to responded (Participants were asked how many years of schooling they had completed, but frequently reported the age that they left education)

±This question was asked differently after Round 1, as there appeared to be some confusion in how to responded (Participants were asked how many years of schooling they had completed, but frequently reported the age that they left education)

In each round 8.7 to 10.1% of respondents reported that the youngest child had a problem with at least one of seeing, hearing, moving or communicating. Problems seeing were most common, followed by communicating and moving (see S1 Tables).

### 3.2 Impacts by nurturing care framework domain

Table 3A summarises the reported impacts in each round across the domains of Nurturing Care.

**Table 3:** Key results by category and survey round.

			ROUND	1	2	3	4	5
category	**Question**		n	774	664	595	529	642
A – Nurturing care domains								
Health	**Missed/delayed…**							
	…**vaccination appointment**… …since start of pandemic^.		% parents/carers of <12m	19.7				
	…in past 30 days		% parents/carers of <12m	–	13.3	7.9	6.3	2.2
	…**growth monitoring…** …since start of pandemic.		%	28.8	–	–	–	–
	…in past 30 days.		%	–	17.0	13.6	7.6	7.0
	…**seeking healthcare for an unwell child**… …since start of pandemic.		%	21.2	–	–	–	–
	…in past 30 days.		%	–	10.7	6.9	7.6	6.1
	**…antenatal appointment…**							
	…since start of pandemic.		%	8.7	–	–	–	–
	…in past 30 days.		%	*–*	2.6	1.2	0.9	1.6
	**…family planning appointment…**							
	…in past 30 days.		%	*–*	5.9	5.4	4.5	5.1
Nutrition	Are you facing any problems with **feeding your child/children at the moment** (either having enough to provide, or with physical feeding)?		% Yes	72.1	46.5	52.1	46.7	46.1
	% (of whole round sample) who report that this is because…							
	…we don’t have enough money.		%	68.9	38.8	46.4	42.5	43.5
	…I am scared to go out.		%	2.8	1.1	0.7	1.5	0.6
	…there is no food available locally.		%	0.3	0.8	0.2	0.0	0.0
	…I breastfeed, and am having problems with breastfeeding		% of those w child < 24m	11.3	5.3	1.6	9.6	9.5
	**Household food security:**							
	Reported skipping a meal because of a lack of money/resources in the last 30 days		% Yes	–	83.4	84.2	77.8	77.1
	Reported not eating for a whole day because of lack of money/resources in the last 30 days		% Yes	–	44.0	38.7	38.3	38.2
Responsive caregiving								
PROVIDER	Used paid childcare in last 3 days		% Yes	1.6	17.6	19.4	18.2	21.2
	Used grandparent provided care			14.2	23.9	24.0	19.8	24.5
	Used sibling provided care			31.2	20.5	21.8	18.2	19.7
Affection	Have you found it more difficult to be affectionate to [name] over the past 30 days?		% Yes	28.3	15.	13.0%	14.5	12.5
Early learning/ education: Family Care Indicators								
	In the past 3 days, did you or anyone else age 15 or over engage in any of the following activities with [name]?							
	Read books		% Yes	32.1	43.6	48.7	47.1	52.5
	Tell stories		% Yes	32.0	37.0	40.4	37.8	44.2
	Sing songs		% Yes	72.4	73.3	72.7	70.3	76.4
	Take outside		% Yes	50.6	67.0	68.0	71.3	68.0
	Name, count, draw		% Yes	39.9	54.3	53.0	55.0	60.4
	Play		% Yes	72.1	76.6	74.6	76.6	81.3
	How many children’s books or picture books do you have for [name]?		% with zero	54.6	38.9	47.5	44	42.2
			% with 0–5	96.5	94.5	97.8	95.6	96.7
			% with >5	3.5	5.5	2.3	4.4	3.3
Security and safety								
NEGLECT	left alone for >1hr for on one or more days in last 7		%	23.5	19.3	12.7	14.9	13.7
		0–5m	%	15.8	17.2	2.6	13.6	2.9
		6–11m	%	15.5	15.9	11.5	13.5	15.8
		12–35m	%	25.0	21.4	13.3	13.7	12.4
		36–59m	%	28.7	18.5	12.5	13.0	11.2
		60m+	%	35.0	21.2	15.6	16.0	16.6
community safety	Has there been any change to how safe, in terms of the risk of violence, your neighbourhood feels since [the start of the pandemic? (R1) | in past 30 days? (R2)]							
			WORSENING	29.9	29.5	31.3	31.2	33.3
			NO CHANGE/unsure	24.1	37.0	43.7	44.2	44.2
			IMPROVEMENT	45.9	33.5	25.0	24.6	22.4
	“Do you think there has been any change in the amount of violence within households (domestic violence) in your community in the past month?” (R2+)							
			% Yes – violence increasing	–	25.6	26.4	24.4	22.4
			% No/don’t know	–	50.7	55.5	56.5	53.1
			% Yes –violence decreasing	–	23.8	18.2	19.1	24.5
		% Yes–increasing (female respondents)		–	25.8	24.6	22.7	22.2
B–Cross-cutting impacts								
	Has how [name of youngest child] spends their time changed…							
	…since Covid started to affect Kenya?		% ‘Yes’	63.4				
	…in the last 30 days?		% ‘Yes’		35.6	20.0	19.0	15.3
Economic effects	Has the type of work you do changed since Covid started to affect Kenya?		% reporting loss of job/work	80.3%				
	Have you lost your job, or changed your work in the past YEAR?		% Yes	–	60.6%	58.8%	45.4%	45.2%
	Have you lost your job, or changed your work in the past MONTH?		% Yes	–	24.7	22.0	16.5	16.8
	How has the amount of money you get changed since start of pandemic?		% reporting earning less	99.7	–	–	–	–
			% reporting earning more	0.1	–	–	–	–
	How has the amount of money you get changed in the past 30days?		% reporting earning less	–	85.4	86.3	80.4	83.8
			% reporting earning more	–	3.5	1.4	2.5	1.7
C–More direct effects of covid:								
	“In the last 30 days, I or a family member have been unwell with COVID symptoms”			1.3	0.6	0.3	0.6	0.3
	Which of the following best describes how you feel about COVID 19?		% My biggest concern	50.1	36.4	39.7	44.2	38.0
			% Very concerned	36.9	40.3	42.2	32.5	29.9
			% Somewhat concerned	10.7	16.7	15.8	19.3	24.5
			% Not at all concerned	2.3	6.6	2.4	4.0	7.6
D–Help received								
	Proportion of sample reporting having received any help in the month before the survey round:		%	70.1	7.5	4.5	4.5	2.6

#### 3.2.1 Health.

Significant disruptions were reported to a broad set of health services in early survey rounds, and although disruptions continued, there was a reduction in the reported incidence over time. In R1, when asked about disruptions to health services since the start of the pandemic in Kenya, the most frequently reported missed or delayed services was child growth monitoring (reported to have been missed/delayed by 28.8% of respondents) followed by missed vaccination appointments (which was reported for 19.7% of children aged under 12 months old) and seeking care for an unwell child (21.2% reported missing this, across all ages of child). In R1 8.7% of parents/carers reported a missed or delayed antenatal appointment since the start of the pandemic in R1.

In subsequent rounds, respondents were asked about disruptions in the previous 30 days. Across these rounds, there is a largely consistent pattern of declining levels of reported disruption round to round. By R5, 7.0% reported having missed/delayed a growth monitoring appointment, 6.1% delaying/avoiding seeking care for an unwell child, and 5.1% having missed a family planning appointment, and 1.6% an antenatal appointment. Only 2.3% of respondents with a youngest child aged under 12 months reported having missed a vaccination appointment in the last 30 days by R5.

#### 3.2.2 Nutrition.

A high proportion of respondents reported problems feeding their child/children in all rounds (R1: 72.1%, R2-5 46.1–52.1%). A large majority of these reported that this was because of having insufficient money (68.9% of respondents reported this in R1), with a much smaller proportion citing being afraid to go out (2.8%) as the reason for their difficulties. Few if any respondents reported food being unavailable locally in any round (0%–0.8%). Amongst respondents with children aged under 24 months, breastfeeding problems were cited by 11.3% in R1, and between 1.6% and 9.6% in subsequent rounds, with no clear trend in over time.

In R2-5 questions from the Food Insecurity Experience Scale (FIES) [20] were included in the survey. In R2, 83% of respondents reported having skipped a meal and 44% reported having not eaten for a whole day due to a lack of money/resources. Reported indicators of food insecurity remained high but dropped slightly in subsequent rounds. By R5, 77% reported having skipped a meal and 38% reported having not eaten for a whole day.

#### 3.2.3 Responsive Care.

In R1, only 1.6% of respondents reported having used paid childcare in the previous 3 days. In subsequent rounds the levels of use of paid childcare increased considerably, for example to 17.6% in R2 and to 21.2% in R5. Box 1 summarizes the frequency of use by age, hours and fees paid for childcare reported. Grandparent provided care was common across all rounds, but was lowest in R1 (14.2%), and highest in R5 (24.5%). Sibling provided care was common in R1 (28.3% reported using it) but declined to 12.5% use by R5.

When asked “Have you found it more difficult to be affectionate to [name] over the past 30 days?” 28.3% said yes in R1, 15.6% in R2 and 12.5% by R5.

**Box 1: Use of Paid Childcare**

Fig 2 illustrates the probability of children of different ages using paid childcare in each round. Older children may have more commonly used childcare in early rounds. However, by R4-R5, other than uncommon childcare use amongst children > 60m, the probability of children using childcare was similar across all other age groups.

Those who reported using paid childcare were additionally asked about how much it cost, which days it was used, and what hours it was reported to be used for. Most (67–87%) of childcare users in all rounds reported attending for 5 or more days each week (S1 Tables). The most common times for using paid childcare were mornings (77–88% of users each round) and afternoons (72–88%), with evening childcare also being frequently used (18–42% of users), but no respondents reporting using paid childcare overnight (S1 Tables). The average spend on childcare was similar across rounds, with the median being KES 50–70 (around 0.5 – 0.7 USD) in all rounds (the mean ranged from 67 KES in R4 to 82 KES in R1)(S1 Tables).

#### 3.2.4 Early learning (and education).

The most commonly reported activities from the Family Care Indicators were playing, singing songs and (especially in subsequent rounds after R1) taking children outside: 72–81% of children were reported to have had a parent or someone over 15 years old playing with them in the previous 3 days; 70–76% reported to have sung songs; and although in R1 only 51% reported having taken their youngest outside in the previous 3 days, in R2-R5 67–71% reported having done so.

Fig 3 is a heatmap illustrating the percentages reporting each Family Care Indicator for each age group for each round. Some activities were reported more frequently with older children (reading, counting/naming/drawing, and telling stories) and singing was more common with younger children. It also appears that there was a modest increase over time in several activities that was not obviously age confounded.

There were few books reported to be in the home; a high proportion of respondents in all rounds reported having no children’s books or picture books (38.9%–54.6%); only 2.3–5.5% reported owning more than 5 books.

#### 3.2.5 Security and safety.

In R1, 23.5% of respondents reported having left their child alone for more than an hour on at least 1 day in the past week. This reduced to 19.3% in R2, and 12.7%–14.9% in R3-5. Although the practice of leaving children alone seemed to be more common with older ages of children, the % of very young children (those aged under 6 months old) reported to be left alone was still high; up to 17% in R2 (Fig 4).

Being left under the care of children was also common, with 44.9% reporting in R1 having left their youngest child under the care of someone aged under 15 years old for more than 1 hour in the past week. In R2, 47.1 reported leaving a child with an under 15-year-old, and 33.5% leaving them with an under 10-year-old. The frequency of being left under the care of a child declined from R1 to R3, and then remained relatively consistent, with 37.1% and 26.9% being left alone with an under 15-year-old and an under 10-year-old respectively by R5.

When asked about community safety “Has there been any change to how safe, in terms of the risk of violence, your neighbourhood feels [since the start of the pandemic – R1/ in the past 30 days – R2-5]?” a mixed picture emerged, and this evolved over time. Across all rounds, up to one third of respondents reported a worsening of community safety (range: 29.5%[R2] to 33.3%[R5]). However in earlier rounds a large proportion (45.9% in R1, 33.5% in R2) of respondents suggested safety had improved. By R5, the largest proportion (44.2%) reported that safety had stayed the same or ‘Don’t know’.

Reported violence towards young children was similar across rounds, ranging between 6.3% in R1 to 4.8% in R4 responding ‘Yes’ to the question “In the past month, has anyone been angry or violent towards your youngest child?”. Amongst children under 2 years-old, the rate varied between 1.3% (R2) and 5.4% (R3) across all 5 rounds.

In rounds 2–5, an additional question asked about perceptions of any change in household/domestic violence; in all but R5, a slightly higher proportion of respondents suggested that domestic violence was increasing (25.6%–24.4% R2-4), than suggested it was falling ((23.8%–19.1% R2-R4). In all rounds around half of respondents replied that it was not changing or ‘Don’t know’. Amongst female respondents (those reporting their relationship to the child was one of being a mother, grandmother or aunt) the rates of increased perceptions of domestic violence were similar to those in the whole sample (25.8%[R2] to 22.2%[R5]).

## 4 Cross-cutting impacts

Table 3B summarises reported cross-cutting impacts, including effects on how young children spent their time and on household income and work. In R1, nearly two thirds (63.4%) reported that the pandemic had led to a change in how their young child spent their time, with the top reported reasons for this being: the closure of childcare; lack of money making childcare unaffordable; the impact of partial reopening of schools; and reductions in contacts with peers/relatives. Over the course of survey rounds, there was a tendency towards lower levels of reported disruption, with only 15.3% reporting a change in the past 30 days by R5, compared to 35.6% reporting this in R2.

Economic impacts of the pandemic were reported to be widespread; in R1 80.3% reported a loss of job/source of income since the start of the pandemic, and 99.7% of respondents reported earning less than in the pre-pandemic period. When asked in subsequent rounds about change in the past 30 days between 80.4% and 86.3% continued to report a reduction in income each round. Very few respondents (0.1–3.5%) reported an increase in earnings in any round.

### 4.1 Reported incidence and concern about Covid-19

When asked about any other impacts of the pandemic, a small proportion of respondents, between 1.3% in R1 and 0.3% in R3, reported that they or a family member had been unwell with Covid-19 symptoms (Table 3C). Levels of concern about Covid-19 were very high initially (with 87% of respondents saying that Covid-19 was their “biggest concern” (50%) or that they were “very concerned” about it (37%)), and although this declined over the course of the data collection period, even by R5, 38% were still saying that Covid-19 was their biggest concern.

### 4.2 Help received

The proportion of people reporting having received any help was significantly higher in R1(70.1%) compared to R2 (7.5%) and later rounds, falling to 2.6% in R5. Most help was reported to have been received in the earlier time periods surveyed, and was in the form of being given information (reported to as received by 41.5% in R1), being given support for their mental wellbeing (for example someone sitting with you, talking with you), (28.8% in R1), and donations of masks (28.6% in R1).

## 4 Discussion

### Principal findings

There are several notable features of the reported impacts of the pandemic on Nurturing Care in slums. Firstly, the impacts were broad in their extent, affecting all components of Nurturing Care. We show that healthcare was radically disrupted, especially earlier in the pandemic. We also identified high levels of reported food and nutrition insecurity. High levels of disruption to responsive caregiving were reported, including to the use of paid childcare. It was common for children, including very young children, to be left alone especially earlier in the pandemic. Secondly, there was considerable change in reported Nurturing Care over time as the pandemic and control measures evolved from an initial strict ‘lockdown’ with closed schools and workplaces to a near-return to normal by the final round of data collection. Underlying all of these was an almost universal experience of a significant economic shock, with almost all respondents reporting a loss of work or income. Strikingly, despite high levels of concern about Covid-19, especially in early rounds, very few study participants reported direct experience of Covid-19 illness in their family in any round.

### Situating these findings within the broader literature

These findings are broadly consistent with other research that has been published on the impacts of the pandemic on the care of young children, albeit with few comparators from informal settings and studies which have tracked impacts over time. For example, health service disruption has been documented through time-series analyses in Kenya which showed that the biggest declines in activity were in outpatient visits and childhood immunisations, with some rebound as the pandemic evolved [21]. Several studies have documented the radical economic shocks precipitated by the pandemic, including globally/regionally [22] and within Kenyan slums particularly, where the pandemic and resulting government policies were reported to have had devastating consequences on the livelihood of slum dwellers who were “left to choose between life and livelihood” [23], “violating” their human right to food [24]. Such findings were replicated in Bangladesh, where the impacts on household economics [25,26] and the mental health of caregivers, generally women [25], were stark. In addition, the quantitative findings presented in this paper are consistent with our qualitative research with parents living in Nairobi slums during the pandemic, which described socio-economic impacts which were deep, protracted, and widely felt, especially amongst women [27,28].

One review that looked at the impacts of the pandemic on Nurturing Care found a bias within the published literature towards quantitative studies in high-income countries and those focused on caregiver mental health [9]. That said, a wide range of UNICEF reviews has documented a broad set of global impacts on children arising from the pandemic, including early learning losses [29], nutrition impacts [30], disruption to child protection [31] and health services [32,33]. Few studies have looked at trends over time as the pandemic evolved, but some have sought to estimate how disruptions to health [34] and broader early childhood adversities precipitated by the pandemic may translate into short- and long-term impacts [35].

By the final round of our data collection, around 1 in 5 children were attending paid childcare regularly. This is lower than another estimate in a different Nairobi slum (Korogocho), in which 46% of employed and 23% of unemployed mothers reported using paid childcare for daytime care for their children aged 1–3 years [11]. This variance could be related to differences in sampling, the ongoing impacts/legacy of the pandemic, differences between the settings or differences in how the question was framed and asked. The fees charged that we found are similar to another recent Kenyan study, and highlight the limited resources in the childcare system [36].

### Unanswered questions and future research

to build on this research it would be valuable to examine how the disruptions noted in this study translate into impacts on child outcomes. In the short-term, this could include child health and development outcomes and then school readiness, but longer term it will be important to understand how the early childhood adversities experienced by this cohort of children, and parents, translates into later life learning, earning and wellbeing. In addition, further analyses and future studies should unpack and better characterise the gendered aspects of the impacts we report, and how this might inform future policy and programming. It is also important and urgent that further implementation research is conducted to develop and test intervention strategies, potentially building on the potential of the childcare platform, to mitigate from some of the disruptions we and others report. Such research should build on long-standing calls for greater emphasis on slums and the people who live in them, and approaches to building resilience in these settings [37] and ought to place particular emphasis on young children’s wellbeing and Rights. Related, these findings underscore the need for better research to characterise the childcare system, including the economics, policies and perspectives of key stakeholders involved, in and beyond Kenya [38,39]. In all of these, the gendered dimensions of care ought to be considered.

### Policy implications

This research suggests a number of ways in which policy and practice can better serve young children growing up in slums or informal settlements. Firstly, as noted by others [24] people, and especially children, growing up in slums deserve a much greater consideration in public including pandemic policy making. The application of blanket policies to very different settings ought to be avoided [23], and in particular the practicalities and impacts of attempting to ‘lockdown’ a slum – where most residents shop daily for necessities, and many rely on daily wages – in particular ought to be better recognised [40]. Secondly, the data presented here and in related studies implies an urgent need for implementation of ambitious and well-resourced post-pandemic interventions to address both the underlying vulnerabilities experienced by young children growing up in slums, and the ways that there were amplified by the pandemic and attempts to control it. Paid childcare – a platform that seems to be widely used yet significantly under-resourced–may provide an intervention opportunity to address some of the risks identified in this research [41]. Finally, noting that many of the risks to the wellbeing of children we predicted early in the pandemic with accompanying calls for mitigation of harms to young children [35], given that these calls seem to have rarely been heeded, it may be that more research and innovation is needed into the structural changes needed to better bring the next generation’s voices and rights into decision making [42].

### Strengths

of this study include its longitudinal design; five survey rounds spread over a year provide insights into temporal changes and trends in the nurturing care of young children in these slums. In addition, the explicit focus on people living in slums provides valuable insights into an often-underrepresented demographic in ECD research [43]. Finally, the multi-domain approach, encompassing all components of nurturing care, offers a broad assessment of the impacts of the pandemic and attempts to control it on young children in these settings.

### Limitations

include a reliance on self-reporting, which may introduce biases or inaccuracies in the data, including both recall bias and the potential for response bias including strategic misrepresentation (if respondents thought that replying in certain ways might lead to allocation of resources to their predicament) [44]. The reliance on a pre-existing database of potential respondents, all of whom had access to a mobile phone, may also have introduced selection bias including an over sampling of better off participants. In addition, the changes in the respondent pool over the study period could affect the consistency of the findings (although efforts were made to account for the risk of age confounding in the analysis of results). The absence of good quality similar baseline data also restricts the analyses that can be completed with our data. Finally, the focus on three slums in Nairobi limits the extrapolation of findings to other settings.

## 5 Conclusion

The early years are a period of both opportunity and vulnerability. This study explored how the care of young children growing up in slums was affected by the Covid-19 pandemic and the control measures brought in to control it. A deep and broad set of impacts across all domains of Nurturing Care were evident. While some of the disruptions appeared to resolve over the five rounds of data collection, others – most notably those related to the economic shocks related to the pandemic – persisted. Provision of paid childcare was effectively suspended in the early part of the pandemic, but usage recovered over time. Collectively, these findings imply an urgent need for greater policy attention being paid to the care of young children growing up in slums, especially at times of crisis.

## Supporting information

S1 Appendix CATI survey instrument. (DOCX)

S2 Appendix Consent script and anticipated ‘frequently asked questions’. (DOCX)

S1 Fig Sankey diagram illustrating flow and loss between survey rounds. (DOCX)

S1 Tables Supplementary tables. (DOCX)
