# Correction: Assessing the impact of Covid-19 on nurturing care in nairobi slums: Findings from 5 rounds of cross-sectional telephone surveys

**DOI:** 10.1371/journal.pgph.0006128

**Published:** 2026-03-25

**Authors:** The PLOS Global Public Health Staff

The word “Nairobi” is misspelled in the article title. The correct title is: Assessing the impact of Covid-19 on nurturing care in Nairobi slums: Findings from 5 rounds of cross-sectional telephone surveys. The correct citation is: Hughes RC, Onyango S, Langat N, Muendo R, Juel R, Kimani-Murage E, et al. (2025) Assessing the impact of Covid-19 on nurturing care in Nairobi slums: Findings from 5 rounds of cross-sectional telephone surveys. PLOS Glob Public Health 5(5): e0003286. https://doi.org/10.1371/journal.pgph.0003286

The publisher apologizes for the error.
