# Correction: Assessing the impact of Covid-19 on nurturing care in nairobi slums: Findings from 5 rounds of cross-sectional telephone surveys

**DOI:** 10.1371/journal.pgph.0006407

**Published:** 2026-04-30

**Authors:** The PLOS Global Public Health Staff

## Notice of Republication

This article was republished on April 20^th^, 2026, to correct an error in the title: the n in Nairobi was erroneously capitalized. Please download this article again to view the correct version. The originally published, uncorrected article and the republished, corrected articles are provided here for reference.

## Supporting information

S1 File Originally published, uncorrected article. (PDF)

S2 File Republished, corrected article. (PDF)
