# Supplementary material for: Assessing the impact of Covid-19 on nurturing care in Nairobi slums: Findings from 5 rounds of cross-sectional telephone surveys
Source: PLOS Glob Public Health. 2025 May 28;5(5):e0003286. doi: 10.1371/journal.pgph.0003286 (PMC12118846; doi:10.1371/journal.pgph.0003286)
Supplement: S1 Appendix — (DOCX) [file pgph.0003286.s001.docx]

**Supplementary Information:**

# S1 Appendix: CATI survey instrument

Following review of Round 1 data quality and free text ‘other’ answers, additional questions were added to: (i) provide additional verification of ages (some ages were being recorded in years not months); (ii) to add family planning to the list of healthcare disruptions specifically asked about; (iii) to collect more detailed information on the provider of childcare throughout the day and costs/timing of any paid childcare uses, and (iv) to add additional questions on household economics and food security.

**R1 Survey Instrument:**

| **No.** | **Domain** | **Script** | **Skips** | **Responses** |  |  |  |
| --- | --- | --- | --- | --- | --- | --- | --- |
| 1 | RESPONDENT | I’d like to start with a couple of questions about you. |  |  |  |  |  |
| 1.1 |  | What year were you born? |  | - [YEAR] - Don’t know   If year 2002 or later, re-confirm eligibility (age >18). If not eligible, thank, and end call. |  |  |  |
| 1.3 |  | What is the highest level of education that you completed? |  | [select 1]   - Never been to school - School up to year: ____ - Some college (including diploma) but not graduate - Graduate - Post-graduate |  |  |  |
| 2 | YOUNGEST CHILD | I’d now like to ask a few questions about your youngest child and what they do day to day. |  |  |  |  |  |
| 2.1 |  | You mentioned the youngest child living with/who lived with you earlier this year is aged <INSERT YOUNGEST AGE FROM SCREENING QUESTION S3a>.  What is their first name? |  | <FIRST NAME YOUNGEST CHILD> |  |  |  |
| 2.2 |  | What is your relationship to <FIRST NAME YOUNGEST CHILD>? |  | [select 1]   - Mother - Father - Grandmother - Grandfather - Aunt - Uncle - Older sibling - Other: specify |  |  |  |
| 2.3 |  | What month and year was <FIRST NAME YOUNGEST CHILD> born? |  | <MONTH and YEAR BORN YOUNGEST CHILD>  *If responses to 2.2 and S3a are inconsistent (computer to automatically note this), probe further and correct. If it turns out not eligible; advise this, thank and end call.* |  |  |  |
| 2.4 |  | Is <FIRST NAME YOUNGEST CHILD> living with you at the moment? |  | - Yes - No |  |  |  |
| 2.5 |  | If No to 2.4.  Where are they living at the moment? | If yes to 2.4 | [Operator to code response to below options; do not read them out - select 1]   - At partner/husband/wife’s separate home - At other relative’s home – local - At other relative’s home – not local (outside of Nairobi) - At a friend/neighbour’s home – local - At a friend/neighbour’s home – not local (outside of Nairobi) - Other: specify |  |  |  |
| 2.6 |  | If No to 2.4.  When did they last live with you? | If yes to 2.4 | [Month]  [Year]  *If not in 2020; re-confirm eligibility* |  |  |  |
| 2.7 |  | If No to 2.4.  Why did <FIRST NAME YOUNGEST CHILD> move there? | If yes to 2.4 | [Operator to code response to below options; do not read them out - select all mentioned]   - Because of COVID-19; worried about risk to child - Because of COVID-19; other: specify - Because it is safer there - Because of my work - Because of my partner’s work - Other: specify |  |  |  |
| 2.8 |  | If Yes to 2.4.  Please can you tell us all of the people who took care of <FIRST NAME YOUNGEST CHILD> for more than an hour during the last **3 days**, not including today? | If No to 2.4 | [Operator to code response to below options; do not read them out - select 1]   - Me - My husband/wife/partner - Child’s grandmother - Child’s grandfather - Child’s sibling(s) - Local friend/neighbour - Nanny - Daycare/childcare/pre-school provider - Other relative – specify - Other friend/neighbour – non-local |  |  |  |
| 2.9 |  | If Yes to 2.4.  Did <FIRST NAME YOUNGEST CHILD> spend more than 1 hour being cared for in any of the following locations in the past **3** **days**?   - at my place of work - at my husband/wife/partner’s place of work - at my husband/wife/partner’s home (if you do not live together) - at a relative’s home - at a relative’s place of work - in my ancestral home/village (not with you) - at a daycare/childcare centre or pre-school - at a friend/neighbour’s home - Other: specify | If No to 2.4 | [Operator to read out list and record responses]   - Y/N/DK - Y/N/DK - Y/N/DK - Y/N/DK - Y/N/DK - Y/N/DK - Y/N/DK - Y/N/DK - Y/N/DK - Y/N/DK - Y- Specify: |  |  |  |
| 2.10 |  | Only if DAYCARE mentioned in 2.4-2.5:  How much do you pay for day care at the moment per day? | If no use of daycare | [Options to allow response per day/week/month]  [KES] |  |  |  |
| 2.10 |  | Only if DAYCARE mentioned in 2.4-2.5:  Does this fee include:   - food for <NAME YOUNGEST CHILD>? - Nappies/daipers | If no use of daycare | - Y/N/DK - Y/N/DK |  |  |  |
| 2.11 |  | Only if DAYCARE mentioned in 2.4-2.5:  For which part or parts of the day does <NAME> normally attend daycare? | If no use of daycare | [Operator to code response to below options; do not read them out - select all that apply]   - Mornings - Afternoons - Evenings - Overnight |  |  |  |
| 2.12 |  | Only if NANNY mentioned in 2.4-2.5:  How much do you pay for your nanny at the moment per day?  Do you provide lodging for your nanny?  Do you provide food for your nanny? | If no use of nanny | [Options to allow response per day/week/month]   - [KES] per day/week/month - Y/N/DK - Y/N/DK |  |  |  |
|  |  | In some questions, I’m interested to know if and how things have changed for you since COVID-19 started to impact on Kenya and your community.  If it is helpful to think of a specific time, you can think of this as being since schools in Kenya were closed because of COVID-19 (in March 2020). |  |  |  |  |  |
| 2.13 |  | Has how <NAME YOUNGEST CHILD> spends their time day to day changed since COVID-19 started to affect you and your community? |  | - No - Yes |  |  |  |
| 2.14 |  | If YES to 2.10:  Please can you tell me how it has changed?  Probe after initial response: Thank you. Are there any other ways it has changed? |  | [Operator to code response to below options; do not read them out - select ALL that are noted]   - My child has moved out of the city - My other child/children’s school has closed, so they can take care of <NAME YOUNGEST CHILD> - My daycare/childcare/pre-school has closed - My work has changed so I am spending more time with <NAME YOUNGEST CHILD> - My work has changed so I am spending LESS time with <NAME YOUNGEST CHILD> - I can no longer afford to pay for daycare/childcare - I have been unable to see older relatives - Seeing fewer peers - Seeing extended family less - Spending less time under care of friend/neighbour - Other – specify: (short text) |  |  |  |
| 2.15 |  | Sometimes adults taking care of children have to leave the house to go shopping, wash clothes, or for other reasons and have to leave young children.  On how many days in the past **seven days** was <NAME YOUNGEST CHILD>:  [A] Left alone for more than an hour? |  | <DAYS WHEN LEFT ALONE>  If ‘None’ record ’0’. If ‘Don’t know’ record ’8’. |  |  |  |
| 2.16 |  | [B] Left in the care of another child less than 10 years old, for more than an hour? |  | <DAYS WHEN LEFT UNDER CARE OF CHILD>  If ‘None’ record ’0’. If ‘Don’t know’ record ’8’. |  |  |  |
| 2.17 |  | In the past 3 days, did you or anyone else age 15 or over engage in any of the following activities with <NAME YOUNGEST CHILD>:   - *Read books or looked at picture books with (****name****)?* - *Told stories to (****name****)?* - *Sang songs to or with (****name****), including lullabies?* - *Took (****name****) outside the home?* - *Played with (****name****)?* - *Named, counted, or drew things for or with (****name****)?*   If ‘Yes’, ask:  Who engaged in this activity with <NAME YOUNGEST CHILD>?  *A foster/step mother or father living in the household who engaged with the child should be coded as mother or father.*  Record all that apply.  ‘No one’ cannot be recorded if any household member age 15 and above engaged in activity with child. |  | \|  \| \| \| \| \| \| --- \| --- \| --- \| --- \| --- \| \|  \| **Mother** \| **Father** \| **Other** \| **No one** \| \| Read books \| A \| B \| X \| Y \| \| Told stories \| A \| B \| X \| Y \| \| Sang songs \| A \| B \| X \| Y \| \| Took outside \| A \| B \| X \| Y \| \| Played with \| A \| B \| X \| Y \| \| Named \| A \| B \| X \| Y \| |  |  |  |
| 2.18 |  | How many children’s books or picture books do you have for <NAME YOUNGEST CHILD>? |  | None 00  Number of children’s books __  Ten or more books 10 |  |  |  |
| 2.19 |  | I am interested in learning about the things that (***name***) plays with when (he/she) is at home.  Does (he/she) play with:  [A] Homemade toys, such as dolls, cars, or other toys made at home?  [B] Toys from a shop or manufactured toys?  [C] Household objects, such as bowls or pots, or objects found outside, such as sticks, rocks, animal shells or leaves? |  | Y N DK  Homemade toys 1 2 8  Toys from a shop 1 2 8  Household objects  or outside objectS…………….……………………1 2 8 |  |  |  |
| 2.20 |  | Have any of the following been missed or delayed because of the COVID-19 epidemic (by you or anyone in your household)?  Vaccination appointment  Child growth check (weight/height measurement)  Seeking healthcare for an unwell child  Antenatal clinic visit  Other healthcare: (specify)  School/nursery enrollment |  | [Operator to read out list and record responses]   - Y/N/DK - Y/N/DK - Y/N/DK - Y/N/DK/NA - ____ - Y/N/DK/NA |  |  |  |
| 2.21 |  | If yes to any of 2.20  What was the reason? | IF NO to 2.20 | [Operator to record which of the following are mentioned; do not read list]   - I am worried about my child being infected - I am not allowed to leave the house because of the lockdown - Someone in the household prevented me from going - I haven’t got enough money to pay for transport - I haven’t got enough money to pay for the health service - The clinic or health service is not operating/a lower capacity - I couldn’t get off work - Other: specify |  |  |  |
| 2.22 |  | Are you facing any problems with feeding your child/children at the moment (either having enough to provide, or with physical feeding)? |  | - Yes - No |  |  |  |
| 2.23 |  | IF YES to 2.22:  What sort of problems are you facing?  Probe after initial response: Thank you. Are there any other problems? | IF NO to 2.22 | [Operator to record which of the following are mentioned; do not read list]   - I breastfeed, and am having problems with breastfeeding - There's not enough good food in the house because we don’t have enough money - There's not enough food in the house because I am scared to go out - There's no transport to get food - I don’t have enough money for transport to get food - No food is being sold or is available near where I live - My child is unwell, restless or irritable and doesn't want to eat - S/he is being fussy about what s/he eats - Other: specify |  |  |  |
| 2.24 |  | Are any of these (issues with feeding) worse since COVID-19 started to affect you and your community? |  | - Yes - No - Not sure |  |  |  |
| 2.25 |  | Which of the following best describes how you feel about COVID-19? |  | [Operator to read out list and record single response chosen by respondent]   - I am **not at all concerned** about COVID-19 - I am **somewhat concerned** about COVID-19 - I am **very concerned** about COVID-19 - COVID-19 is **my biggest concern** |  |  |  |
| 2.26 |  | Has there been any change to how safe, in terms of the risk of violence, your neighborhood feels since the nationwide curfew came in due to COVID-19? |  | - No - Yes – it feels safer - Yes – it feels less safe - Not sure |  |  |  |
| 2.27 |  | In the past month, has anyone been angry and violent towards you?  [Alternative framing – consider in pre-testing: Do you think there has been any change in the amount of violence within households in your community in the past month? – options: Yes – increasing violence; No; Yes – falling violence] |  | - No - Yes - Prefer not to answer |  |  |  |
|  |  |  |  | *IF YES: offer to signpost to Police/Domestic Abuse resource centre; offer to share telephone number now, and/or to send follow up SMS* |  |  |  |
| 2.28 |  | In the past month, has anyone been angry and violent towards <NAME YOUNGEST CHILD>? |  | - No - Yes - Prefer not to answer |  |  |  |
|  |  |  |  | *IF YES: offer to signpost to Childline Kenya resource centre; offer to share telephone number now, and/or to send follow up SMS* |  |  |  |
| 2.29 |  | Have you found it more difficult to be affectionate to <NAME YOUNGEST CHILD> over the past month?  [Consider re-ordering, to after 2.25] |  | - Yes - No |  |  |  |
| 2.30 |  | [IF YES to 2.29]  Why do you think this may be? | IF NO to 2.26 | [Operator to record which of the following are mentioned; do not read list]   - I am stressed and irritable - I feel hopeless, I am depressed and withdrawn - My child is crying a lot and is difficult to comfort - There is a lot of tension in our house - Other: specify |  |  |  |
| 2.31 |  | Has COVID-19 had an impact on <NAME YOUNGEST CHILD> in any other way that we haven’t discussed? |  | - No - Yes: Specify [short text] |  |  |  |
| 2.32 |  | Are there any other ways in which COVID-19 is affecting you or your family which you think are important? |  | [Operator to record which of the following are mentioned; do not read list]   - I or a family member have been unwell with COVID symptoms - I or a family member have been unwell with something else, but have not sought healthcare - Seeing friends less - Seeing family less - Other: specify |  |  |  |
| 2.33 |  | Have you received any help over the past month? |  | - No - Not sure - Yes |  |  |  |
| 2.34 |  | IF YES to 2.33  Have you received any of the following forms of help over the past month?   - Donated food - Donations for my child, like clothes - Donated medicine - Given me information - Donated masks, soap, sanitizer, gloves - Lent or given me money (with conditions/requirements) - Lent or given me money (with NO conditions/requirements) - Given support for my mental distress (sat with you, prayed with you) - Educational support - Other help? | If No/not sure to 2.30 | [read list]   - Y/N/DK - Y/N/DK - Y/N/DK - Y/N/DK - Y/N/DK - Y/N/DK - Y/N/DK - Y/N/DK - Y/N/DK - Y/N/DK - Other:   Thank you. I’ve noted that. |  |  |  |
| 3 | HH SES | Finally, I’d like to ask a few questions about your household and your work. |  |  |  |  |  |
| 3.1 |  | What is the main way that you get money at the moment? |  | [select 1]   - Doing paid domestic work - Doing factory work - Selling in the market - Trading; buying, transporting, selling - Working in food stalls, bars - Construction work - Remittances (money sent to you) - No source of income - Get from other household members - Get from friends   Other: |  |  |  |
| 3.2 |  | Has the type of work you do changed since COVID-19 started to affect you and your community? |  | - Yes - No - Prefer not to say |  |  |  |
| 3.3 |  | IF YES to 3.2  What was the main way you got money **before** COVID started to affect you and your work? |  | [Select all that apply]   - Doing paid domestic work - Doing factory work - Selling in the market - Trading; buying, transporting, selling - Working in food stalls, bars - Construction work - Remittances - No source of income - Get from other family members - Get from friends   Other: |  |  |  |
| 3.4 |  | Has the amount of money you get changed since COVID-19 started to affect you and your community? |  | - Yes - No - Prefer not to say |  |  |  |
| 3.4 |  | IF YES to 3.4  How has the amount of money you get changed since COVID-19 started to affect you and your community? |  | - I am getting less money - I am getting more money - Prefer not to say |  |  |  |
| 3.5 |  | Can I ask you, do your household own any of the following possessions? |  | \| **Item** \| **Y/N** \| **Item** \| **Y/N** \| **Item** \| **Y/N** \| \| --- \| --- \| --- \| --- \| --- \| --- \| \| Car or truck \|  \| Sewing machine \|  \| Torch \|  \| \| Motorcycle \|  \| Electric iron \|  \| Kerosene lamp \|  \| \| Bicycle \|  \| Fan \|  \| Kerosene stove \|  \| \| Refrigerator \|  \| Mobile phone \|  \| Wall clock \|  \| \| Television \|  \| Gas Cooker \|  \| Mattress \|  \| \| Radio/Stereo \|  \| Sofa \|  \| Blankets \|  \| \| DVD Player \|  \| Table \|  \| Bed \|  \| |  |  |  |
| 4 |  | THANK YOU. We have finished the survey. I will arrange for KES150 of airtime to be sent to your cellphone.  Would you like it sent to the number I called you on today, or a different number? |  | - Same - Different:   - Specify number:   - Network: |  |  |  |
| 4.1 |  | May we call you back in around 1 month to ask some more questions? |  | Y/N |  |  |  |
| 4.2 |  | Would you like me to send a follow up SMS message with some contact details for people who can provide information on:   - COVID-19 in Kenya? - Child health/wellbeing/safety? - How to contact the researchers at the African Population Health Research Centre? |  | Y/N  Y/N  Y/N |  |  |  |
|  |  | Thank you for your time today. Goodbye. |  |  |  |  |  |

**R2+ Survey questions:**

| **No.** | **Domain** | **Script** | **Skips** | **Responses** |
| --- | --- | --- | --- | --- |
| 1 | RESPONDENT | I’d like to start with a couple of questions about you. |  |  |
| 1.1 |  | What month and year were **you** born? |  | - [YEAR] - Don’t know   If year 2002 or later, re-confirm eligibility (age >18). If not eligible, thank, and end call. |
| 1.1 |  | So this means **you** are ___ years old; is this correct? |  | - Y/N   Cross-check with age screening above; do not proceed until age confirmed and clear, and at least year of birth recorded. |
| 1.1 |  | **How old were you** when you stopped education (school or college/university?) |  | [AGE] or Don’t know |
| 1.1 |  | **What grade** of school did you complete |  | - INSERT OPTIONS BASED ON KENYAN SYSTEM - DK |
| 2 | YOUNGEST CHILD | I’d now like to ask a few questions about <NAME YOUNGEST CHILD> and what they do day to day. |  |  |
| 2.1 |  | Are they a boy or a girl? |  | - M - F - Prefer not to answer |
| 2.3 |  | At different ages, care of children can be quite different, so we are keen to know as accurately as possible how old <FIRST NAME YOUNGEST CHILD> is?  What year and month where they born? |  | <MONTH and YEAR BORN YOUNGEST CHILD>  *If responses to 2.2 and S3a are inconsistent (computer to automatically note this), probe further and correct. If it turns out not eligible; advise this, thank and end call.* |
| 1.1 |  | So this means <FIRST NAME YOUNGEST CHILD> is (__ years and) ___months old today; is this correct?  AGE CALCULATED FROM Responses to 2.3 |  | - Y/N   Cross-check with age screening above; do not proceed until age confirmed and clear, and at least year of birth recorded, and confirmed eligibility (age >18yrs). |
| 2.4 |  | Is <FIRST NAME YOUNGEST CHILD> living with you at the moment? |  | - Yes - No |
| 2.5 |  | If No to 2.4.  Where are they living at the moment? | If yes to 2.4 | [Operator to code response to below options; do not read them out - select 1]   - At partner/husband/wife’s separate home - At other relative’s home – local - At other relative’s home – not local (outside of Nairobi) - At a friend/neighbour’s home – local - At a friend/neighbour’s home – not local (outside of Nairobi) - Other: specify |
| 2.6 |  | If No to 2.4.  When did they last live with you? | If yes to 2.4 | [Month]  [Year]  *If not in 2020; re-confirm eligibility* |
| 2.7 |  | If No to 2.4.  Why did <FIRST NAME YOUNGEST CHILD> move there? | If yes to 2.4 | [Operator to code response to below options; do not read them out - select all mentioned]   - Because of COVID-19; worried about risk to child - Because of COVID-19; other: specify - Because it is safer there - Because of my work - Because of my partner’s work - Other: specify |
|  |  | Does ${nameyoungest} have any difficulty doing any of the following? Je ${nameyoungest} ana changa moto ya kufanya ya fwatayo? |  |  |
|  |  | - Seeing (even with glasses, if you wear them) Kuona(hata kama anatumia miwani) |  | Y/N/DN |
|  |  | - Hearing (even with a hearing aid, if you wear one) Kusikia(hata kama anatumia chombo ya kumsaidia kusikia) |  | Y/N/DN |
|  |  | - Moving/Walking/crawling Kujisongesha/Kutembea/Kutambaa |  | Y/N/DN |
|  |  | - in communicating in usual language, including sign language (i.e. understanding others or being understood by others) Kuwasiliana kwa lugha ya kawaida, pamoja na lugha ya ishara( yaani kuelewa wengine au wengine kumuelewa) |  | Y/N/DN |
| 2.8 |  | If Yes to 2.4.  We are interested to know **who** <NAME YOUNGEST> spends time with day to day.  Please can you tell us all of the people who took care of <FIRST NAME YOUNGEST CHILD> **for any period of time, even a few minutes,** during the last **3 days**, not including today? | If No to 2.4 | [Operator to code response to below options; do not read them out - select all that are mentioned]   - Child’s mother - Child’s father - Child’s aunt - Child’s uncle - Child’s grandmother - Child’s grandfather - Child’s sibling(s) - Local friend/neighbour/someone I work with - Nanny - Daycare/childcare/pre-school provider - Don’t know - Other– specify |
| 2.8 |  | If Yes to 2.4.  Please can you tell us all of the people who took care of <FIRST NAME YOUNGEST CHILD> **for more than one hour** during the last **3 days**, not including today? | If No to 2.4 | [Operator to code response to below options; do not read them out - select all that are mentioned]   - Child’s mother - Child’s father - Child’s aunt - Child’s uncle - Child’s grandmother - Child’s grandfather - Child’s sibling(s) - Local friend/neighbour/someone I work with - Nanny - Daycare/childcare/pre-school provider - Don’t know - Other– specify |
| 2.9 |  | If Yes to 2.4.  We are also interested to learn **where** <NAME YOUNGEST> spends his/her time.  Did <FIRST NAME YOUNGEST CHILD> spend more than 1 hour in any of the following locations in the past **3** **days**?   - at my husband/wife/partner’s home (if you do not live together) - at his/her mother’s workplace - at his/her father’s workplace - at a daycare/childcare centre or pre-school - at school - around the neighbourhood (not at home) - Travelling – on a matatu or bodaboda or taxi etc. - at a relative’s home - at a relative’s workplace - at a friend/neighbour’s home - at a friend/neighbour’s workplace - in my ancestral home/village (not with you) - church - Other: specify | If No to 2.4 | [Operator to read down list and record Y/N/DK responses for each]   - Y/N/DK for each |
| 2.8 |  | If Yes to 2.4.  **I’m interested to know how these change as the day goes on. I’d like you to think about yesterday, or the last weekday if yesterday was a weekend day,**  From when you woke up, please can you tell me **who, if anyone,**  was responsible for/looking after <NAME YOUNGEST> during each hour of the day from when the child wakes up: | If No to 2.4 | [Operator to code response to below options; do not read them out - select all that apply for each period; the aim being to record who, if anyone, is responsible for the child for what the parent considers a significant amount of time in that period]   \|  \| 6-7am \| 7-8am \| 8-9am \| 10-11am \| 11am-12pm \| 1-2pm \| 2-3pm \| 3-4pm \| 4-5pm \| 5-6pm \| 7pm+ and overnight \| \| --- \| --- \| --- \| --- \| --- \| --- \| --- \| --- \| --- \| --- \| --- \| --- \| \| Child’s mother \|  \|  \|  \|  \|  \|  \|  \|  \|  \|  \|  \| \| Child’s father \|  \|  \|  \|  \|  \|  \|  \|  \|  \|  \|  \| \| Child’s aunt \|  \|  \|  \|  \|  \|  \|  \|  \|  \|  \|  \| \| Child’s uncle \|  \|  \|  \|  \|  \|  \|  \|  \|  \|  \|  \| \| Child’s grandmother \|  \|  \|  \|  \|  \|  \|  \|  \|  \|  \|  \| \| Child’s grandfather \|  \|  \|  \|  \|  \|  \|  \|  \|  \|  \|  \| \| Child’s sibling(s) \|  \|  \|  \|  \|  \|  \|  \|  \|  \|  \|  \| \| Local friend/neighbour \|  \|  \|  \|  \|  \|  \|  \|  \|  \|  \|  \| \| Nanny \|  \|  \|  \|  \|  \|  \|  \|  \|  \|  \|  \| \| Daycare/childcare/pre-school provider \|  \|  \|  \|  \|  \|  \|  \|  \|  \|  \|  \| \| Nobody – looking after self \|  \|  \|  \|  \|  \|  \|  \|  \|  \|  \|  \| \| Other \|  \|  \|  \|  \|  \|  \|  \|  \|  \|  \|  \| \| Don’t know  [only 1 response allowed for this time period this is checked] \|  \|  \|  \|  \|  \|  \|  \|  \|  \|  \|  \| |
| 2.9 |  | If Yes to 2.4.  **Again, thinking about the last weekday:**  **Where** did <FIRST NAME YOUNGEST CHILD> spend for each of these times during these times **on the last week weekday**? | If No to 2.4 | [Operator to read out list and record responses; more than one allowed for each time period, unless ‘don’t know’ is selected]   \|  \| 6-7am \| 7-8am \| 8-9am \| 10-11am \| 11am-12pm \| 1-2pm \| 2-3pm \| 3-4pm \| 4-5pm \| 5-6pm \| 7pm+ and overnight \| \| --- \| --- \| --- \| --- \| --- \| --- \| --- \| --- \| --- \| --- \| --- \| --- \| \| at my husband/wife/partner’s home (if you do not live together) \|  \|  \|  \|  \|  \|  \|  \|  \|  \|  \|  \| \| at his/her mother’s workplace \|  \|  \|  \|  \|  \|  \|  \|  \|  \|  \|  \| \| at his/her father’s workplace \|  \|  \|  \|  \|  \|  \|  \|  \|  \|  \|  \| \| at a daycare/ childcare centre or pre-school \|  \|  \|  \|  \|  \|  \|  \|  \|  \|  \|  \| \| at school \|  \|  \|  \|  \|  \|  \|  \|  \|  \|  \|  \| \| around the neighbourhood (not at home) \|  \|  \|  \|  \|  \|  \|  \|  \|  \|  \|  \| \| Travelling – on a matatu or bodaboda or taxi etc. \|  \|  \|  \|  \|  \|  \|  \|  \|  \|  \|  \| \| at a relative’s home \|  \|  \|  \|  \|  \|  \|  \|  \|  \|  \|  \| \| at a relative’s workplace \|  \|  \|  \|  \|  \|  \|  \|  \|  \|  \|  \| \| at a friend/neighbour’s home \|  \|  \|  \|  \|  \|  \|  \|  \|  \|  \|  \| \| at a friend/neighbour’s workplace \|  \|  \|  \|  \|  \|  \|  \|  \|  \|  \|  \| \| in my ancestral home/village (not with you) \|  \|  \|  \|  \|  \|  \|  \|  \|  \|  \|  \| \| Church \|  \|  \|  \|  \|  \|  \|  \|  \|  \|  \|  \| \| don’t know  [only 1 response allowed for this time period this is checked] \|  \|  \|  \|  \|  \|  \|  \|  \|  \|  \|  \| \| Other \|  \|  \|  \|  \|  \|  \|  \|  \|  \|  \|  \| |
| 2.10 |  | Only if DAYCARE mentioned in 2.4-2.5:  How many days per week does <NAME YOUNGEST> attend daycare? | If no use of daycare | [Number of days] – 1-7 range  DK - |
| 2.11 |  | Only if DAYCARE mentioned in 2.4-2.5:  For which part or parts of the day does <NAME> normally attend daycare? | If no use of daycare | [Operator to code response to below options; do not read them out - select all that apply]   - Mornings - Afternoons - Evenings - Overnight |
| 2.10 |  | Only if DAYCARE mentioned in 2.4-2.5:  How much do you pay for day care at the moment **per day**? | If no use of daycare | [KES per day; **if respondent reports per week or month, enumerator to divide by number of days attending and check back with respondent**] |
| 2.10 |  | Only if DAYCARE mentioned in 2.4-2.5:  Does this fee include:   - food for <NAME YOUNGEST CHILD>?   Does this fee include:   - Nappies/diapers | If no use of daycare | - Y/N/DK - Y/N/DK |
| 2.12 |  | Only if NANNY mentioned in 2.4-2.5:   - How much do you pay for your nanny at the moment per day? - Do you provide lodging for your nanny? - Do you provide food for your nanny? | If no use of nanny | [Options to allow response per day/week/month]   - [KES] per day – enumerator to calculate daily pay if paid weekly/monthly and verify - Y/N/DK - Y/N/DK |
|  |  | In some questions, I’m interested to know if and how things have changed in the last month (30 days). |  |  |
| 2.13 |  | Has how <NAME YOUNGEST CHILD> spends their time day to day changed in the last month (30 days)? |  | - No - Yes |
| 2.14 |  | If YES to 2.10:  Please can you tell me how it has changed?  Probe after initial response: Thank you. Are there any other ways it has changed? |  | [Operator to code response to below options; do not read them out - select ALL that are noted]   - My child has moved out of the city - My other child/children’s school has **closed**, so they can take care of him/her - My other child/children’s school has **opened**, so they can take care of him/her - My daycare/childcare/pre-school has **closed** - My daycare/childcare/pre-school has **openend** - My work has changed so I am spending **more** time with him/her - My work has changed so I am spending **LESS** time with him/her - I can no longer afford to pay for daycare/childcare - I have been unable to see older relatives - Spending **less** time with/seeing fewer peers - Spending **more** time with his/her peers - Seeing extended family less - Spending less time under care of friend/neighbour - Spending **less** time at home - Spending **more** time at home - Other – specify: (short text) |
| 2.15 |  | Sometimes adults taking care of children have to leave the house to go shopping, wash clothes, or for other reasons and have to leave young children.  On how many days in the past **seven days** was <NAME YOUNGEST CHILD>:  [A] Left alone for more than an hour? | If not currently living with respondent | <DAYS WHEN LEFT ALONE>  If ‘None’ record ’0’. If ‘Don’t know’ record ’8’. |
| 2.16 |  | [B] Left in the care of another child less than 10 years old, for more than an hour? | If not currently living with respondent | <DAYS WHEN LEFT UNDER CARE OF CHILD>  If ‘None’ record ’0’. If ‘Don’t know’ record ’8’. |
| 2.16 |  | [B] Left in the care of another child less than 15 years old, for more than an hour? | If not currently living with respondent | <DAYS WHEN LEFT UNDER CARE OF CHILD>  If ‘None’ record ’0’. If ‘Don’t know’ record ’8’. |
| 2.17 |  | In the past 3 days, did you or anyone else age 15 or over engage in any of the following activities with <NAME YOUNGEST CHILD>:   - *Read books or looked at picture books with (****name****)?* - *Told stories to (****name****)?* - *Sang songs to or with (****name****), including lullabies?* - *Took (****name****) outside the home?* - *Played with (****name****)?* - *Named, counted, or drew things for or with (****name****)?*   If ‘Yes’, ask:  Who engaged in this activity with <NAME YOUNGEST CHILD>?  *A foster/step mother or father living in the household who engaged with the child should be coded as mother or father.*  Record all that apply.  ‘No one’ cannot be recorded if any household member age 15 and above engaged in activity with child. |  | NOTE: Record all that apply.  ‘No one’ cannot be recorded if any household member age 15 and above engaged in activity with child.   \|  \| \| \| \| \| \| --- \| --- \| --- \| --- \| --- \| \|  \| **Mother** \| **Father** \| **Other** \| **No one** \| \| Read books \| A \| B \| X \| Y \| \| Told stories \| A \| B \| X \| Y \| \| Sang songs \| A \| B \| X \| Y \| \| Took outside \| A \| B \| X \| Y \| \| Played with \| A \| B \| X \| Y \| \| Named \| A \| B \| X \| Y \| |
| 2.18 |  | How many children’s books or picture books do you have for <NAME YOUNGEST CHILD>? |  | None 00  Number of children’s books __  Ten or more books 10 |
| 2.19 |  | I am interested in learning about the things that (***name***) plays with when (he/she) is at home.  Does (he/she) play with:  [A] Homemade toys, such as dolls, cars, or other toys made at home?  [B] Toys from a shop or manufactured toys?  [C] Household objects, such as bowls or pots, or objects found outside, such as sticks, rocks, animal shells or leaves? |  | Y N DK  Homemade toys 1 2 8  Toys from a shop 1 2 8  Household objects  or outside objectS…………….……………………1 2 8 |
| 2.20 |  | Have any of the following been missed or delayed for <NAME YOUNGEST> in the past month (30 days?)   - Vaccination appointment - Child growth check (weight/height measurement) - Seeking healthcare because unwell - Other healthcare: (specify) - School/nursery enrollment |  | [Operator to read out list and record responses]   - Y/N/DK - Y/N/DK - Y/N/DK - ____ - Y/N/DK/NA |
|  |  | If yes to missed vaccine appointment:  Do you know what vaccine it was you missed?  If yes, what vaccination was it?  Have you been able to rearrange that vaccination? |  | - Y/N - Freetext: ____ - Y/N/DK |
| 2.21 |  | If yes to any of 2.20  What was the reason? | IF NO to 2.20 | [Operator to record which of the following are mentioned; do not read list]   - I am worried about my child being infected - I am worried about my child being quarantined or taken away from me - I am not allowed to leave the house because of the lockdown - Someone in the household prevented me from going - I haven’t got enough money to pay for transport - I haven’t got enough money to pay for the health service - The clinic or health service is not operating/a lower capacity - I couldn’t get off work - School was closed/not operating normally - Other: specify |
| 2.20 |  | Have you yourself missed or delayed any of the following in the past month (30 days?)   - Seeking healthcare because unwell - Antenatal clinic visit - Family planning/contraception appointment - Other healthcare: (specify) |  | [Operator to read out list and record responses]   - Y/N/DK/NA - Y/N/DK/NA - Y/N/DK/NA - Y/N   ____ |
| 2.21 |  | If yes to any of these  What was the reason? | IF NO to above | [Operator to record which of the following are mentioned; do not read list]   - I am worried about me or my child being infected - I am worried about my child being quarantined or taken away from me - I am not allowed to leave the house because of the lockdown - Someone in the household prevented me from going - I haven’t got enough money to pay for transport - I haven’t got enough money to pay for the health service - The clinic or health service is not operating/a lower capacity - I couldn’t get off work - Other: specify |
| 2.22 |  | Are you facing any problems with feeding your child/children at the moment (either having enough to provide, or with physical feeding)? |  | - Yes - No |
| 2.23 |  | IF YES to 2.22:  What sort of problems are you facing?  Probe after initial response: Thank you. Are there any other problems? | IF NO to 2.22 | [Operator to record which of the following are mentioned; do not read list]   - I breastfeed, and am having problems with breastfeeding - There's not enough good food in the house because we don’t have enough money - There's not enough food in the house because I am scared to go out - There's no transport to get food - I don’t have enough money for transport to get food - No food is being sold or is available near where I live - My child is unwell, restless or irritable and doesn't want to eat - S/he is being fussy about what s/he eats - Other: specify |
| 2.24 |  | Have any of these issues with feeding got better, worse or stayed the same in the past month (30days)? |  | - Better - Worse - Stayed the Same - Don’t know |
| 2.25 |  | Which of the following best describes how you feel about COVID-19? |  | [Operator to read out list and record single response chosen by respondent]   - I am **not at all concerned** about COVID-19 - I am **somewhat concerned** about COVID-19 - I am **very concerned** about COVID-19 - COVID-19 is **my biggest concern** |
| 2.26 |  | Has there been any change to how safe, in terms of the risk of violence, your neighborhood feels since the nationwide curfew came in due to COVID-19? |  | - No - Yes – it feels safer - Yes – it feels less safe - Not sure |
| 2.27 |  | Do you think there has been any change in the amount of violence within households (domestic violence) in your community in the past month? |  | No;  Yes – increasing violence  Yes – falling violence |
|  |  |  |  | *IF YES: offer to signpost to Police/Domestic Abuse resource centre; offer to share telephone number now, and/or to send follow up SMS* |
| 2.28 |  | In the past month, has anyone been angry and violent towards <NAME YOUNGEST CHILD>? |  | - No - Yes - Prefer not to answer |
|  |  |  |  | *IF YES: offer to signpost to Childline Kenya resource centre; offer to share telephone number now, and/or to send follow up SMS* |
| 2.29 |  | Have you found it more difficult to be affectionate to <NAME YOUNGEST CHILD> over the past month?  [Consider re-ordering, to after 2.25] |  | - Yes - No |
| 2.30 |  | [IF YES to 2.29]  Why do you think this may be? | IF NO to 2.26 | [Operator to record which of the following are mentioned; do not read list]   - I am stressed and irritable - I feel hopeless, I am depressed and withdrawn - My child is crying a lot and is difficult to comfort - There is a lot of tension in our house - Other: specify |
| 2.32 |  | Are there any other ways in which, **in the last month (30 days)**, COVID-19 is affecting you or your family which you think are important? |  | [Operator to record which of the following are mentioned; do not read list]   - I or a family member have been unwell with COVID symptoms - I or a family member have been unwell with something else, but have not sought healthcare - I or a family member have had to self-isolate - Seeing friends less - Seeing family less - Travel restrictions - Economic impacts/hardship - Curfew limiting my work - Police brutality - Rising teen pregnancies - Other: specify |
| 2.33 |  | Have you received any help over the past month? |  | - No - Not sure - Yes |
| 2.34 |  | IF YES to 2.33  Have you received any of the following forms of help over the past month?   - Donated food - Donations for my child, like clothes - Donated medicine - Given me information - Donated masks, soap, sanitizer, gloves - Lent or given me money (with conditions/requirements) - Lent or given me money (with NO conditions/requirements) - Given support for my mental distress (sat with you, prayed with you) - Educational support - Other help? | If No/not sure to 2.30 | [read list]   - Y/N/DK - Y/N/DK - Y/N/DK - Y/N/DK - Y/N/DK - Y/N/DK - Y/N/DK - Y/N/DK - Y/N/DK - Y/N/DK - Other:   Thank you. I’ve noted that. |
| 3 | HH SES | Finally, I’d like to ask a few questions about your household and your work. |  |  |
| 3.1 |  | What is the **main** way that you get money at the moment? |  | [select 1]   - Doing paid domestic work - Doing factory work - Selling in the market - Trading; buying, transporting, selling - Working in food stalls, bars - Construction work - Remittances (money sent to you) - No source of income - Get from other household members - Get from friends - Transport worker - bodaboda, matatu, de - Tailor - Teacher - Casual worker - sector unspecified - Hairdresser/barber - Security worker/guard - Mechanic - National hygiene programme Kazi mtaani   Other: |
| 3.2 |  | Have you lost your job, or changed your work in the past **year**? |  | - Yes - No - Prefer not to say |
| 3.2 |  | Have you lost or changed your work in the past **month** (30days)? |  | - Yes - No - Prefer not to say |
| 3.3 |  | IF YES to 3.2  If yes, are you working more, less or the same number of hours each week? |  | - I am working fewer hours each week than I was a month ago - I am working more hours each week than I was a month ago - I am working the same number of hours each week than I was a month ago - Prefer not to say |
| 3.3 |  | IF YES to 3.2  If yes, are you earning more, less or the same? |  | - I am getting less money than I was a month ago - I am getting more money than I was a month ago - I am getting the same amount of money as a month ago - Prefer not to say |
| 4 |  | THANK YOU. We have finished the survey. I will arrange for KES150 of airtime to be sent to your cellphone.  Would you like it sent to the number I called you on today, or a different number? |  | - Same - Different:   - Specify number:   - Network: |
| 4.1 |  | May we call you back in around 1 month to ask some more questions? |  | Y/N |
| 4.2 |  | Would you like me to send a follow up SMS message with some contact details for people who can provide information on:   - COVID-19 in Kenya? - Child health/wellbeing/safety? - How to contact the researchers at the African Population Health Research Centre? |  | Y/N  Y/N  Y/N |
|  |  | Thank you for your time today. Goodbye. |  |  |
