# Supplementary material for: Assessing the impact of Covid-19 on nurturing care in Nairobi slums: Findings from 5 rounds of cross-sectional telephone surveys
Source: PLOS Glob Public Health. 2025 May 28;5(5):e0003286. doi: 10.1371/journal.pgph.0003286 (PMC12118846; doi:10.1371/journal.pgph.0003286)
Supplement: S2 Appendix — (DOCX) [file pgph.0003286.s002.docx]

# S2 Appendix : consent script and anticipated ‘frequently asked questions’

***Introduction and eligibility screening:***

| **No.** | **Script** | **Response and actions.** |
| --- | --- | --- |
|  | Good morning/afternoon/evening. My name is <NAME>, and I am a researcher from Busara. We are conducting a survey to find out how people who live in this area are managing during the COVID-19 pandemic. We are especially interested to learn about the care of young children. |  |
|  | May I ask; some initial questions to see if you are eligible to take part in the survey, and is it ok if we record this part of the call? (I will tell you when we will stop recording). | Y/N    If no, thank and end call. |
| S1 | Where do you normally live? | NAME of Location  [Survey tool to automatically verify if this is a slum area; If not, thank, and inform not eligible; end call. |
| S2 | How old are you? | [YRS]  If No (aged <18yrs) thank, and inform not eligible; end call. |
| S3 | Do you have, or are you looking after, any children? (Alternative: Are you the parent or guardian of any children?) | Y/N  If no, thank and inform not eligible; end call. |
| S3a | If YES;  How old are each of them, starting with the youngest. | [AGE CHILD1], [AGE CHILD2], [AGE CHILD3], etc.  If any children <5 years old; continue.  If no child/children aged <5 years, thank, and inform not eligible; end call. |
| S3c | If Yes to having any children U5 in Q S3a:  Is the youngest child (last born), living with you now? | Y/N – IF YES, eligible; INVITE TO PARTICIPATE  If NO, continue to S3d |
| S3d | If No to S3c:  Have they, any child aged under 5yrs, been living with you at any point since the start of 2020? | Y/N – IF YES, eligible; INVITE TO PARTICIPATE  If no, thank and inform not eligible; end call. |

*[NOTE: above information will not be retained; will only be used for screening inclusion criteria; simple ‘eligible’ or not will be recorded on master database]*

***Consent:***

| **No.** | **Script** | **Response and actions.** |
| --- | --- | --- |
| C1 | You are eligible to take part in this survey, as you live in this area, and have a child who is less than 5 years old. I would like to tell you more about the survey so you can decide if you want to take part? Is that OK? | Y/N  If not interested, thank and end call. |
|  | This study is led by researchers from Kenya and the UK. We want to learn how you and your child/children are doing at the moment. We will ask about your household, your work and how your child/children is/are cared for, including whether this has been affected by COVID-19. The answers will be used to build an understanding of how COVID-19 is affecting people in this area.  The survey will take around 15-20 minutes, and if you complete it you will receive 150KES of airtime to compensate you for your time. Participation is voluntary and you are free to stop the survey at any point, including part way through.  You and around 500 others were selected at random from a list of phone numbers of people who are on Busara’s list of phone numbers of people who have previously given their permission to be contacted for telephone research. and who live in Nairobi.  If you agree to participate your name, phone number and other personal details will not be shared with anyone. We will store your name so we can call you again in the future if you give us permission. Your answers, with all your personal details removed, and the answers of all the other participants may be shared with other researchers.  Results of the research, but not any of your personal details, will be shared in research reports or papers and with people who make decisions about child health and development policy in Kenya.  At the end of this survey, we will also ask you if you would mind us calling you back in 1 month to ask another set of questions.  Do you have any questions? | Y/N |
|  | FAQs + answers:   - Why do you want to talk to me?   - We want to learn about you and your family, including how the coronavirus epidemics affecting your life. This is so that we can try to make sure that those who are trying to design programmes to help people like you understand the issues you face in your daily life. We are especially interested in people who are responsible for young children, as children can be especially affected by events around them. - Will I be paid for my answers?   - If you complete the survey, you will receive KES 150 of airtime and time to compensate you for your time and battery use. - What happens if I change my mind about taking part?   - If you decide to take part, you are free to change your mind at any point, and for any reason. You can just say, and we will not contact you again, and we will delete any answers you have already given.   - Who are you? And who are you working for? My name is X. I work for a group called Busara. We are a research organisation who run telephone surveys. For this project, we are working with researchers from universities in Kenya, The African Population Health Research Centre and the UK (the London School of Hygiene and Tropical Medicine). - Can you give me information about the virus?   - I’m afraid I cannot give you information myself, but I can help to direct you to reliable sources of up to date information. Would you like me to send a text message with some telephone numbers and websites? - Can you help me? I am struggling.   - I’m sorry to hear that. I’m afraid I cannot help directly. I can send you some information about ways to access help though by SMS? Would you like me to send a message with ways to contact organisations who are providing some support? - Whom can I call on further information about the survey?   - You can contact researchers at the African Population Health Research Center who can provide more information about the survey. I can give you their telephone number, and I will also send a SMS message after this call with their telephone number. - I have another question not listed here:   - You can contact researchers at the African Population Health Research Center who can provide more information about the survey. I can give you their telephone number, and I will also send a SMS message after this call with their telephone number. |  |
| C2 | [After answering questions:]  Would you like to take part in the research? | - Yes – now àQuestions - Yes - but, please call me back. àSchedule call for later date/time - No àThank and end call. |

[All of above to be audio recorded, and available for audit as required].
