# Supplementary material for: Assessing the impact of Covid-19 on nurturing care in Nairobi slums: Findings from 5 rounds of cross-sectional telephone surveys
Source: PLOS Glob Public Health. 2025 May 28;5(5):e0003286. doi: 10.1371/journal.pgph.0003286 (PMC12118846; doi:10.1371/journal.pgph.0003286)
Supplement: S1 Fig — (DOCX) [file pgph.0003286.s003.docx]

# S1 Fig: Sankey diagram illustrating flow and loss between survey rounds


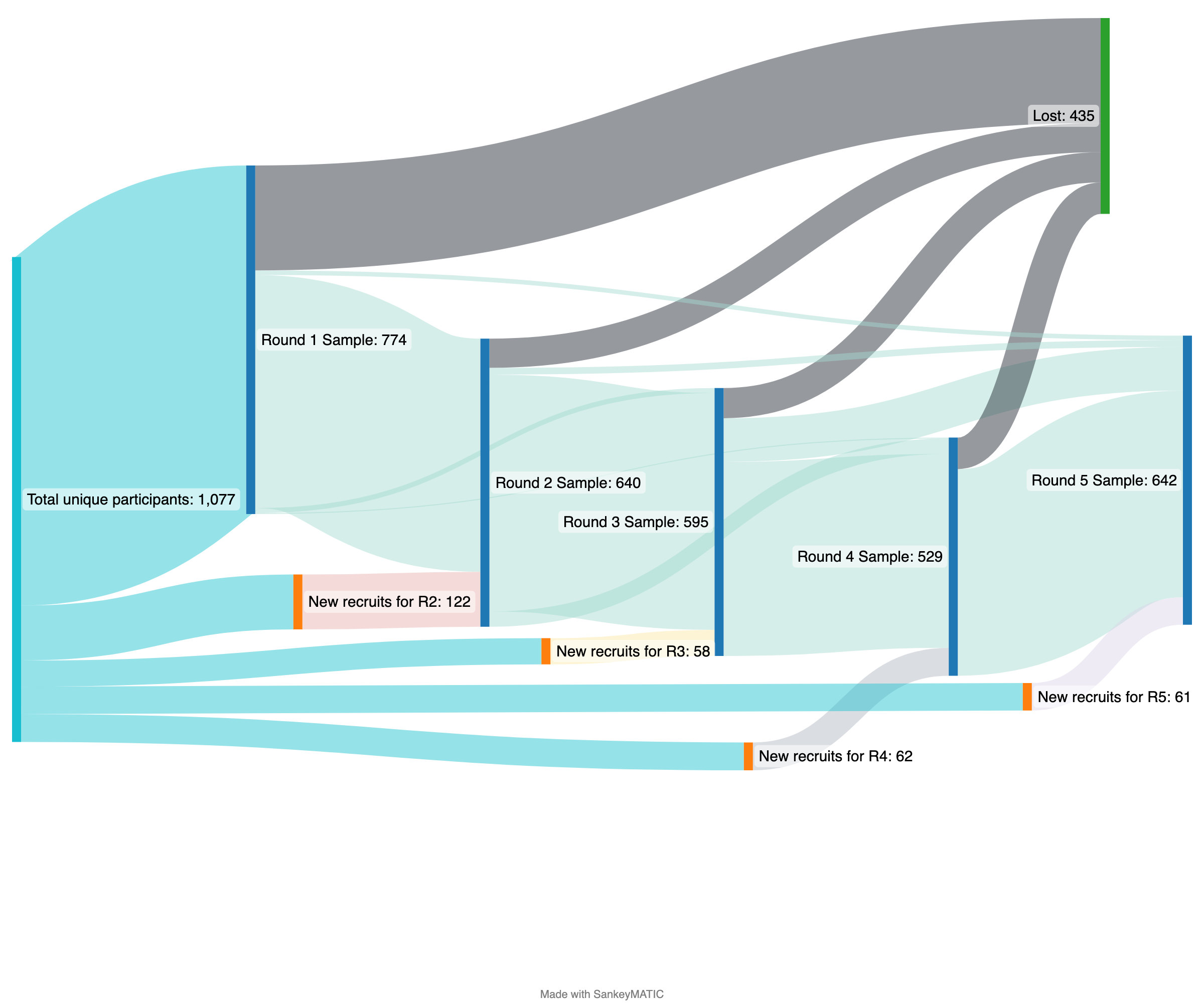


# Supplementary Appendix 3: Supplementary tables

### Supplementary table 1: % reached on each call attempt by round

|  | **R1** | **R2** | **R3** | **R4** | **R5** |
| --- | --- | --- | --- | --- | --- |
| **Median time to complete survey - minutes** | 27 | 34 | 23 | 20 | 17 |
| **% Reached on 1^st^ call attempt** | - | 86 | 92 | 95 | 95 |

### *Notes: Additional questions were added after R1. HH asset questions were only asked on first entry into study. Data on number of attempts unavailable in R1 because of how data was initially coded in SurveyCTO.*

### Supplementary table 2: number of rounds completed by respondents.

279 respondents completed all 5 rounds (197 completed 4 rounds; 131 three rounds; 134 two rounds; 336 one round)

| **Number of rounds completed by respondent:** | **n** | **% of the 1077 unique respondents** |
| --- | --- | --- |
| **5** | 279 | 25.9 |
| **4** | 197 | 18.3 |
| **3** | 131 | 12.2 |
| **2** | 134 | 12.4 |
| **1** | 336 | 31.2 |

### Supplementary table 3: The relationships between respondents and the youngest child they were reporting on (across all rounds)

| **Relationship to youngest child** | **n** | **% of the 1077 unique respondents** |
| --- | --- | --- |
|  |  |  |
| Mother | 555 | 51.5 |
| Father | 382 | 35.5 |
| Aunt | 26 | 2.4 |
| Grandmother | 84 | 7.8 |
| Uncle | 10 | 0.9 |
| Grandfather | 5 | 0.5 |
| Older sibling | 2 | 0.3 |
| Other: specify | 13 | 1.2 |
|  |  |  |
| Total | 1,077 |  |

###

### Supplementary table 4: Disabilities/problems that were reported amongst children across all rounds

| **% of 1077 total respondents who reported their child had difficulty…** | **n** | **% of the 1077 unique respondents** |
| --- | --- | --- |
| **…seeing** | 36 | 3.3 |
| **…communicating** | 30 | 2.8 |
| **…moving** | 29 | 2.7 |
| **…hearing** | 17 |  |

###

### Supplementary table 5: frequency (days of week) children were reported to attend paid childcare by round

| **Number of days/ week users attend childcare** | | **1** | **2** | **3** | **4** | **5** | **6** | **7** | **5+ days** |  | **% 5 or more days (in the round)** |
| --- | --- | --- | --- | --- | --- | --- | --- | --- | --- | --- | --- |
| **Survey Round** | n |  |  |  |  |  |  |  |  |  |  |
| **R2** | 39 | 1 | 5 | 2 | 5 | 19 | 4 | 3 | 26 |  | 66.7% |
| **R3** | 50 | 0 | 0 | 3 | 3 | 37 | 6 | 1 | 44 |  | 88.0% |
| **R4** | 53 | 0 | 0 | 6 | 6 | 30 | 9 | 2 | 41 |  | 77.4% |
| **R5** | 67 | 3 | 0 | 2 | 4 | 51 | 6 | 1 | 58 |  | 86.6% |

### Supplementary table 6: time of day children were reported to attend paid childcare by round

| **% of childcare users attending in:** | **Morning** | **Afternoon** | **Evening** | **Overnight** |
| --- | --- | --- | --- | --- |
| **R2** | 76.9% | 84.6% | 17.9% | 0.0% |
| **R3** | 88.0% | 78.0% | 42.0% | 0.0% |
| **R4** | 83.0% | 71.7% | 30.2% | 0.0% |
| **R5** | 88.1% | 88.1% | 28.4% | 0.0% |

###

### Supplementary table 7: average spend on childcare by round

|  |  | **Spend per day (KES)** |  |
| --- | --- | --- | --- |
| **Survey Round** | n | Median (mean) | Range |
| **R2** | 39 | 70 (82) | 10-250 |
| **R3** | 50 | 50 (70) | 30-300 |
| **R4** | 53 | 50 (81) | 15-250 |
| **R5** | 67 | 50 (67) | 20-150 |
